# Nitric oxide production by tumour tissue: impact on the response to photodynamic therapy

**DOI:** 10.1054/bjoc.2000.1157

**Published:** 2000-06

**Authors:** M Korbelik, C S Parkins, H Shibuya, I Cecic, M R L Stratford, D J Chaplin

**Affiliations:** Cancer Imaging Department, British Columbia Cancer Agency, 601 West 10th Avenue, Vancouver BC, Canada; Gray Laboratory Cancer Research Trust, Northwood, Middlesex, UK

**Keywords:** photodynamic therapy, nitric oxide, ischaemia-reperfusion injury, mouse tumour models, tumour blood flow, nitric oxide synthase inhibitors

## Abstract

The role of nitric oxide (NO) in the response to Photofrin-based photodynamic therapy (PDT) was investigated using mouse tumour models characterized by either relatively high or low endogenous NO production (RIF and SCCVII vs EMT6 and FsaR, respectively). The NO synthase inhibitors N^ω^-nitro- L -arginine (L-NNA) or N^ω^-nitro- L -arginine methyl ester (L-NAME), administered to mice immediately after PDT light treatment of subcutaneously growing tumours, markedly enhanced the cure rate of RIF and SCCVII models, but produced no obvious benefit with the EMT6 and FsaR models. Laser Doppler flowmetry measurement revealed that both L-NNA and L-NAME strongly inhibit blood flow in RIF and SCCVII tumours, but not in EMT6 and FsaR tumours. When injected intravenously immediately after PDT light treatment, L-NAME dramatically augmented the decrease in blood flow in SCCVII tumours induced by PDT. The pattern of blood flow alterations in tumours following PDT indicates that, even with curative doses, regular circulation may be restored in some vessels after episodes of partial or complete obstruction. Such conditions are conducive to the induction of ischaemia-reperfusion injury, which is instigated by the formation of superoxide radical. The administration of superoxide dismutase immediately after PDT resulted in a decrease in tumour cure rates, thus confirming the involvement of superoxide in the anti-tumour effect. The results of this study demonstrate that NO participates in the events associated with PDT-mediated tumour destruction, particularly in the vascular response that is of critical importance for the curative outcome of this therapy. The level of endogenous production of NO in tumours appears to be one of the determinants of sensitivity to PDT. © 2000 Cancer Research Campaign


*British Journal of Cancer* (2000) **82**(11), 1835–1843
© 2000 Cancer Research Campaign
DOI: 10.1054/ bjoc.2000.1157, available online at http://www.idealibrary.com on IDEAL®

### Nitric oxide production by tumour tissue: impact on the response to photodynamic therapy

**M Korbelik[1], CS Parkins[2], H Shibuya[1], I Cecic[1], MRL Stratford[2] and DJ Chaplin[2]**

[1]Cancer Imaging Department, British Columbia Cancer Agency, 601 West 10th Avenue, Vancouver BC, Canada; [2]Gray Laboratory Cancer Research Trust, Northwood, Middlesex, UK

**Summary** The role of nitric oxide (NO) in the response to Photofrin-based photodynamic therapy (PDT) was investigated using mouse tumour models characterized by either relatively high or low endogenous NO production (RIF and SCCVII vs EMT6 and FsaR, respectively). The NO synthase inhibitors $N^{\omega}$-nitro-L-arginine (L-NNA) or $N^{\omega}$-nitro-L-arginine methyl ester (L-NAME), administered to mice immediately after PDT light treatment of subcutaneously growing tumours, markedly enhanced the cure rate of RIF and SCCVII models, but produced no obvious benefit with the EMT6 and FsaR models. Laser Doppler flowmetry measurement revealed that both L-NNA and L-NAME strongly inhibit blood flow in RIF and SCCVII tumours, but not in EMT6 and FsaR tumours. When injected intravenously immediately after PDT light treatment, L-NAME dramatically augmented the decrease in blood flow in SCCVII tumours induced by PDT. The pattern of blood flow alterations in tumours following PDT indicates that, even with curative doses, regular circulation may be restored in some vessels after episodes of partial or complete obstruction. Such conditions are conducive to the induction of ischaemia-reperfusion injury, which is instigated by the formation of superoxide radical. The administration of superoxide dismutase immediately after PDT resulted in a decrease in tumour cure rates, thus confirming the involvement of superoxide in the anti-tumour effect. The results of this study demonstrate that NO participates in the events associated with PDT-mediated tumour destruction, particularly in the vascular response that is of critical importance for the curative outcome of this therapy. The level of endogenous production of NO in tumours appears to be one of the determinants of sensitivity to PDT. © 2000 Cancer Research Campaign

**Keywords:** photodynamic therapy; nitric oxide; ischaemia-reperfusion injury; mouse tumour models; tumour blood flow; nitric oxide synthase inhibitors

Nitric oxide (NO) has become recognized as a major effector molecule in a diverse array of physiologic and pathologic processes (Bredt and Snyder, 1994; Schmidt and Walter, 1994). It has also become evident that this gas radical, produced by many cells in the human body, not only controls important functions in tumour progression, but may have a major influence on the outcome of cancer therapies, particularly those that are mediated by increased generation of reactive oxygen species (oxidative stress) (Jenkins et al, 1995; Xie et al, 1995; Parkins et al, 1995; Tozer and Everett, 1997*a*; Hirst and Flitney, 1997).

Photodynamic therapy (PDT), used for the treatment of various types of cancer (Dougherty et al, 1998), induces a strong oxidative stress and triggers the vascular-mediated response with a massive neutrophil recruitment (Krosl et al, 1995; Gollnick et al, 1997), and these events are prone to be highly sensitive to NO mediation (Moilanen et al, 1993; Schmidt and Walter, 1994; Hirst and Flitney, 1997). In tumours producing high levels of NO, the PDT-induced reduction in tumour blood flow, vascular occlusion and consequent ischaemia may be diminished, while the inflammatory reaction triggered by PDT may be suppressed (Korbelik et al, 1998). This could result from the following effects of NO:

*Received 27 July 1999*
*Revised 4 January 2000*
*Accepted 2 February 2000*

*Correspondence to:* M Korbelik

- Acts as a potent vasodilator.
- Prevents platelet aggregation and adhesion to the endothelium.
- Suppresses the aggregation of accumulated inflammatory neutrophils.
- Inhibits the expression of leukocyte adhesion molecules and hence the adhesion and extravasation of circulating leukocytes.
- Averts mast cell degranulation (Schmidt and Walter, 1994; Vanhoutte, 1987; Kubes et al, 1991).

On the other hand, elevated NO levels may maintain vessel dilation during PDT light treatment, which can diminish the decrease in tumour oxygenation and sustain in this way the oxygen-dependent generation of phototoxic damage (Korbelik et al, 1998). Additionally, NO increases vascular permeability and consequent vascular leakage, which are characteristic occurrences in PDT-treated vasculature (Dougherty et al, 1998). The NO-sensitive processes that unfold after the termination of photodynamic light treatment include:

- Ischaemia-reperfusion injury, where NO can have a protective role.
- Apoptosis of tumour cells, which can be stimulated by NO.
- Development of the immune reaction against the treated tumour, where NO has immunoregulatory functions (Korbelik et al, 1998; Dougherty et al, 1998).

The levels, as well as main cellular sources of endogenously produced NO, vary considerably among solid human and animal tumours (Tozer and Everett, 1997*b*; Parkins et al, 1995). Since NO acts as a vasodilator, its generation may be upregulated in some tumours to compensate for deficient blood supply. Indeed, NO appears to act in the signalling cascade for tumour neovascularization (Jenkins et al, 1995). In a preliminary report, we have suggested that endogenous NO production may represent an important determinant in the response of tumours to PDT (Korbelik et al, 1997).

Marked changes in tumour NO levels may be expected to occur after photodynamic treatment. An increase in the generation of NO was observed following PDT treatment of tumour cells in vitro (Gupta et al, 1998). This was attributed to the enhanced expression of the constitutive form of nitric oxide synthase (NOS), observed to peak at 5 min post-PDT and return to normal levels within 60 min. The increased activity of this $Ca^{2+}$-regulated NOS isoform, which could be tumour type- and PDT photosensitizer-dependent, may be triggered by the PDT-induced release of intracellular calcium stores (Ochsner, 1997). Moreover, the interaction of PDT-based oxidative stress with cellular signal transduction pathways leads to the activation of nuclear transcription factors (Dougherty et al, 1998), which may also result in the upregulation of genes encoding NOS. On the other hand, activated inflammatory cells accumulated in PDT-treated tumours may be responsible themselves for the release of NO (Cecic and Korbelik, 1999; McCall et al, 1989; Mehta et al, 1991; Evans et al, 1996). In contrast, a decreased release of NO can be expected if its main source is impaired by cytotoxic damage inflicted by PDT to cells largely responsible for endogenous NO production (e.g. endothelial cells in tumour vasculature) (Gilissen et al, 1993).

From the above consideration it is clear that the NO levels in tumours and their modulation may considerably impact the curative result of PDT. In the present study, we have investigated the effect of NOS inhibitors on the PDT response of mouse tumours characterized by a different production of endogenous NO.

### MATERIALS AND METHODS

### Tumour models

Squamous cell carcinoma SCCVII (Suit et al, 1985), as well as fibrosarcomas RIF-1 (Twentyman et al, 1980) and FsaR (Volpe et al, 1985) were implanted into syngeneic C3H/HeN mice, while mammary sarcoma EMT6 (Rockwell et al, 1972) was implanted into syngeneic BALB/c mice. For experiments, the tumours were inoculated subcutaneously in lower dorsal region of 7–9 week old female mice. The exception was blood flow experiments, for which the tumours were implanted in the dorsal side of hind left footpads. Details of maintenance and implantation of these tumours have been reported earlier (Korbelik and Krosl, 1996*a*).

### Chemicals

All drugs were purchased from Sigma Chemical Co. (St. Louis, MO, USA). The NOS inhibitors used were $N^{\omega}$-nitro-L-arginine (L-NNA) (Sigma N-5501) and $N^{\omega}$-nitro-L-arginine methyl ester (L-NAME) (Sigma N-5751). The inactive analogue $N^{\omega}$-nitro-D-arginine methyl ester (D-NAME) (Sigma N-4770) was also tested. These arginine analogues were administered at 20 mg $kg^{-1}$ (0.1 ml per 20 g body weight). Superoxide dismutase (SOD) (Sigma S-5639), a manganese-containing enzyme (EC 1.15.1.1), was administered at 450 U per mouse. The same SOD dose was used in a previous work (Parkins et al, 1995). All these compounds were dissolved in 5% dextrose solution and injected intravenously.

### Analysis of NO production using tumour explants

The exact procedure has been described previously (Parkins et al, 1995). Briefly, the excised tumours were weighed, finely minced and incubated in small Petri dishes containing nitrate-free Minimal Essential Medium Eagle (Sigma M-4655) supplemented with 10% FBS (Hyclone Laboratories, Inc., Logan, UT). Aliquots of culture supernatants collected at different time-points after incubation at 37°C in a humidified $CO_2$-air incubator were analysed by high-performance ion chromatography (HPIC) for the concentration of the NO metabolites nitrite and nitrate (Stratford et al, 1997). All the samples collected in the present study showed no significant accumulation of nitrite (NO is oxidized to nitrite first and subsequently to nitrate if haemoglobin or other serum proteins are present). At least four identical tumour explant cultures were used for each determination. The size of these tumours was similar to that used for PDT (see below).

In some experiments, the production of NO was assessed using a modified NOS assay (Rees et al, 1995). In this case, 0.5 μCi of L-[$^{14}$C]arginine (278 mCi $mmol^{-1}$) purchased from Amersham Life Science (Buckinghamshire, UK) was added to tumour explant medium during 1 h incubation at 37°C. NOS activity was determined from the conversion of L-[$^{14}$C]arginine to L-[$^{14}$C]citrulline using a kit produced by Cayman Chemical Company (Ann Arbor, MI, USA).

### PDT treatment

One week after inoculation, tumours reached 5–6 mm in largest diameter and the host mice were administered Photofrin (porfimer sodium, QLT PhotoTherapeutics, Inc., Vancouver, Canada) at 10 mg $kg^{-1}$ i.v. The light treatment was performed 24 h later with doses ranging from 75–180 J $cm^{-2}$, depending on the tumour model. Specially designed lead holders were used for immobilizing the mice during PDT illumination without anaesthesia. The tumours and 1 mm of surrounding normal skin were treated superficially with 630 ± 10 nm light delivered from a tunable light source based on a 1 kW xenon bulb (Model A5000; Photon Technology International, Inc.) through a 5 mm core diameter liquid light guide (2000A; Luminex, Munich, Germany). The power density at the treatment area was 60–70 m W $cm^{-2}$.

### Evaluation of tumour response

After treatment with PDT and/or other agents, mice were observed for tumour regrowth every second day until 90 days post-PDT, at which time mice showing no sign of tumour were considered cured. Each treatment group consisted of eight mice. In the case of treatment with NOS inhibitors only (no PDT), changes in tumour volumes were determined by measuring with a caliper the lesion's three orthogonal diameters.

### Measurement of tumour blood flow

The procedure based on laser Doppler flowmetry was identical to that described by Durand and LePard (1994) and was performed in

 *© 2000 Cancer Research Campaign*

Dr Durand's laboratory. Unanaesthetized mice were immobilized exposing their tumour-bearing leg; tumour size was as described for PDT treatment. Blood flow in superficial tumour regions was measured using a laser Doppler flowmeter (Laserflo Perfusion Monitor, Model BPM 403; TSI, Inc., St Paul, Minnesota, USA), which registers tissue microvascular flow continuously and non-invasively with a spatial resolution of approximately 1 $mm^3$ (Shepherd et al, 1987). The contribution of the superficial skin was found not to obscure tumour-related blood flow changes measured by this instrument (Durand and LePard, 1994). The needle probe with a 0.7 mm tip diameter was placed directly on the skin above the tumour. A winged needle infusion set connected to a syringe containing NOS inhibitor solution was installed into the tail vein of mice before the onset of blood flow measurement. The drug was injected as a bolus (0.1 ml per 20 g body weight) after 30 min recording pre-treatment blood flow. The values for relative blood flow were derived from the number and velocity of moving red blood cells, normalized to the mean pre-treatment value. During the PDT treatment, the laser Doppler flowmetry was stopped and then resumed immediately after the termination of PDT illumination, by carefully repositioning the needle probe on the tumour precisely at the pre-treatment site.

Statistical analysis of the tumour response data was performed using the long-rank test and other data were analysed using Student's *t*-test.

### RESULTS

A previous report (Parkins et al, 1995) has shown that the intrinsic production of NO can considerably vary with different types of mouse tumours. This is also exemplified in Figure 1, which depicts the results of NO production measurement in four mouse tumour models. Explants of freshly excised tumour tissue were incubated ex vivo for 6–24 hours and the oxidized metabolites of NO (nitrite and nitrate) released in the culture medium were analysed by HPIC. Since no significant accumulation of nitrite was detected, the production of NO was assessed based on the increase of nitrate in the explant medium. The results show that RIF and SCCVII tumours are more active producers of NO than FsaR and EMT6 tumours. For instance, RIF tumours produced approximately four-fold more NO than EMT6 tumours. The presence of NO synthase inhibitor L-NNA (1 mM) in the explant medium suppressed the production of NO by these tumours, as illustrated with the SCCVII explants (Figure 1). An alternate method of NO production measurement, a modified version of NOS assay based on $^{14}$C-labelled L-arginine described by Rees et al (1995), produced equivalent results. The NOS activity (pmol $hour^{-1}$ $mg^{-1}$ protein ± SD) in SCCVII, RIF, EMT6 and FsaR tumours was 8.36 ± 1.19, 13.41 ± 8.66, 2.56 ± 1.07, and 0.69 ± 0.44, respectively.

As alluded to above, local levels of NO may influence the response of tumours to therapeutic modalities such as PDT. This was tested by examining the effect of a single treatment with NOS inhibitors L-NNA and L-NAME on the response of 'low' (FsaR and EMT6) and 'high' NO producing tumours (SCCVII and RIF) to Photofrin-based PDT. The effect of a single injection of L-NNA or L-NAME at doses used in these experiments showed no significant effect on tumour growth. An example is depicted in Figure 2, which shows that L-NNA produced an apparent but statistically insignificant temporary retardation of the growth of SCCVII tumours.

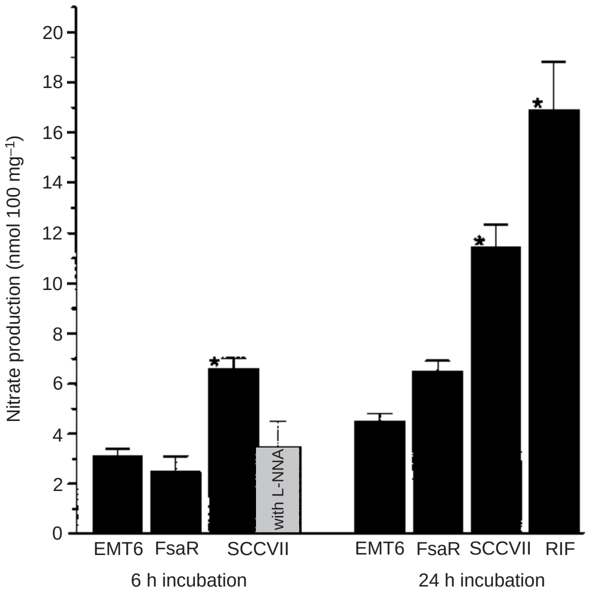

**Figure 1** The production of NO by mouse tumours measured during *ex-vivo* explant culture. Subcutaneously growing EMT6, FsaR, SCCVII and RIF tumours were excised and cultured ex vivo for either 6 or 24 h. The production of NO in tumours was determined from aliquots of culture supernatants analysed for its oxidized product, nitrate. The NOS inhibitor L-NNA (1 mM) was added to some SCCVII explant cultures. Significantly greater nitrate was produced by SCCVII and RIF tumours compared with EMT6 and FsaR tumours (Bars, SD; * = $P$ < 0.005).

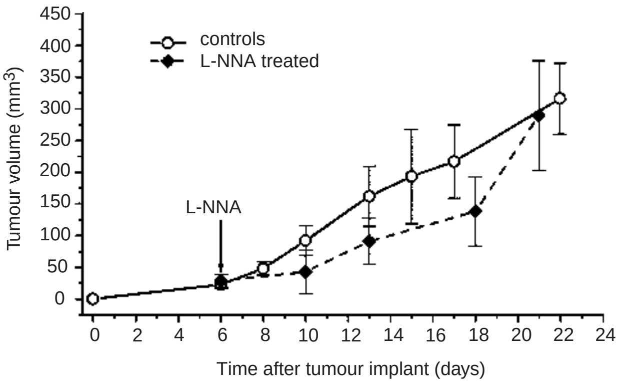

**Figure 2** The effect of L-NNA on growth of subcutaneous SCCVII tumours. The drug was administered intravenously (20 mg $kg^{-1}$) at 6 days post-tumour implant. Changes in tumour volumes were monitored for 16 days after L-NNA injection.

The photosensitizer and light dose combination used for the treatment of SCCVII tumours produced their complete initial ablation, but all the tumours re-grew within 2–3 weeks (Figure 3). In contrast, in mice that received L-NAME or L-NNA (20 mg $kg^{-1}$ i.v.) immediately after the termination of photodynamic light treatment, 40–50% of tumours remained non-palpable over 90 days after treatment, which qualifies as tumour cure. Similar results were obtained with RIF tumours, as illustrated for the PDT plus L-NAME combination in Figure 3. While the chosen PDT-only treatment was not curative, over 60% of RIF tumours were cured when L-NAME was injected immediately after PDT. As shown in the same graph, delaying the L-NAME administration to 30 min or 24 h after PDT abrogated the beneficial effect on the tumour

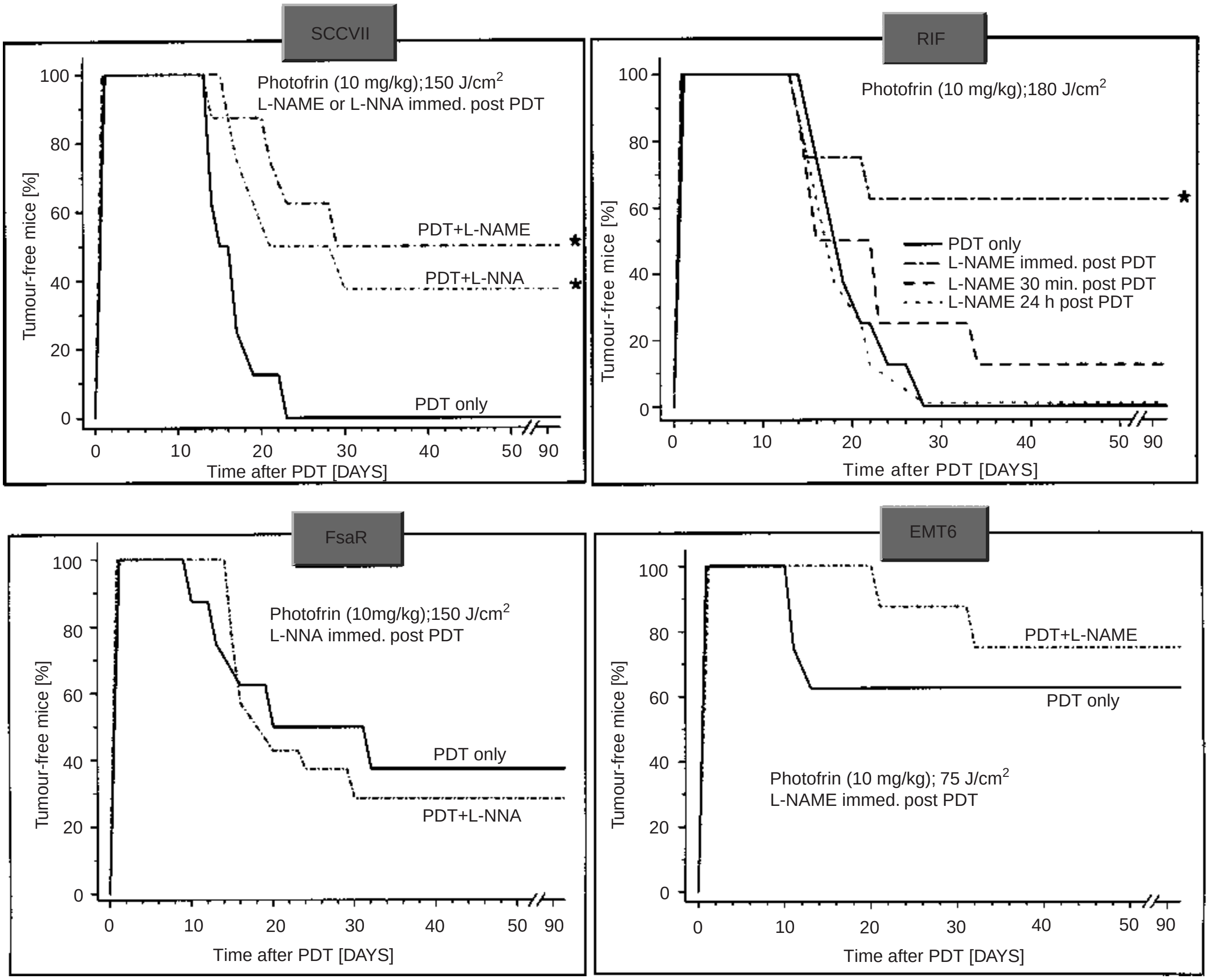

**Figure 3** The effects of L-NNA and L-NAME on the response of various tumour models to PDT. Tumours growing in syngeneic mice were treated with light (150 J $cm^{-2}$ for SCCVII and FsaR, 180 J $cm^{-2}$ for RIF, and 75 J $cm^{-2}$ for EMT6) at 24 h after Photofrin administration (10 mg $kg^{-1}$ i.v.). L-NNA or L-NAME were injected immediately after light treatment (both at 20 mg $kg^{-1}$ i.v.). The mice were thereafter monitored for signs of tumour growth or its absence. Eight tumour-bearing mice were treated in each experimental group. * = $P < 0.05$ (compared to PDT only).

response. The administration of NOS inhibitors before the PDT light treatment diminished the curative effect of PDT (Korbelik and Krosl, 1996*b*), presumably because of reduced tumour oxygenation during light treatment (Wood et al, 1994).

The adjuvant treatment with NOS inhibitors produced no therapeutic benefit with PDT-treated FsaR and EMT6 tumours. The examples for L-NNA with FsaR tumours and for L-NAME with EMT6 tumours (the drugs injected immediately after PDT), depicted in Figure 3, show that no significant difference in tumour cures was obtained between PDT-only and PDT plus L-NNA/L-NAME treatment groups. The results with these two tumour models indicate that they exhibit greater intrinsic sensitivity to PDT than RIF and SCCVII tumours.

The NOS inhibitors L-NNA and L-NAME are known to induce the inhibition of tumour blood flow (Andrade et al, 1992; Horsman et al, 1996), but no evidence is available on the relevance of the level of intrinsic NO production in tumours for the expression of this effect. The effects of these two drugs on tumour blood flow in the four models used in this study are shown in Figure 4. The results of blood flow measurement, using laser Doppler flowmetry, in four representative individual tumours growing in mice injected with L-NNA are shown on the left. It is evident that this drug induced a different response in RIF tumours than in FsaR tumours, which were growing in the same strain of mice. The blood flow in RIF tumours started to decrease within 5 min after L-NNA injection, dropped to 20–50% of normal values during the next 10–15 min, and showed little, if any, signs of recovery at 60 min post-drug administration. In contrast, the blood flow of low NO producing FsaR tumours was either moderately decreased or showed no obvious changes. At 30 min after the L-NNA injection, the relative blood flow levels in RIF and FsaR tumours were $0.40 \pm 0.22$ and $0.81 \pm 0.18$ (means ± SD, $n = 4$), respectively.

The effects of L-NAME on the blood flow in RIF, SCCVII and EMT6 tumours, presented in this case as average values from measurements in 3–4 tumours, are shown in the right side graph of Figure 4. The inhibitory effect of this drug was clearly evident with the blood flow in RIF and SCCVII tumours, showing a similar pattern as depicted for L-NNA in RIF tumours. However, in parallel with the situation shown for L-NNA with FsaR

 *© 2000 Cancer Research Campaign*

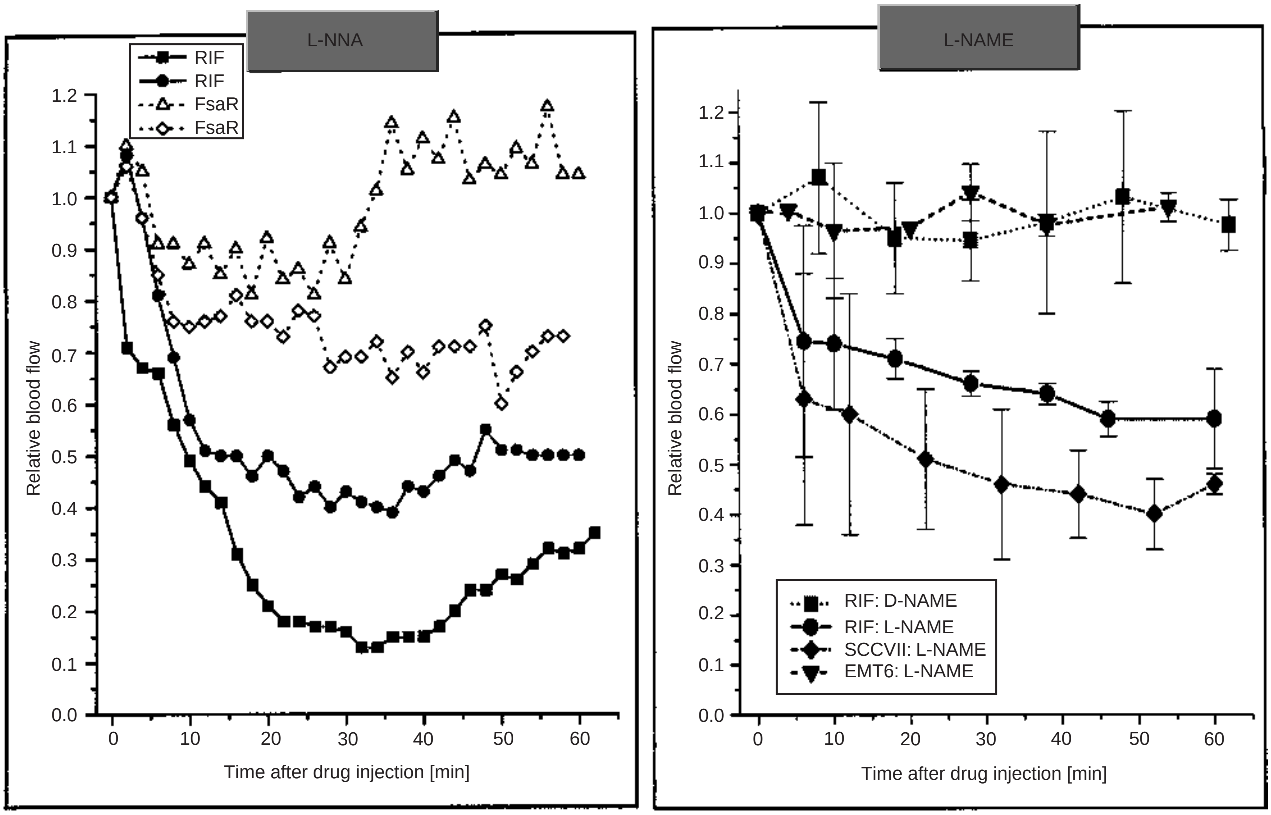

**Figure 4** The effect of L-NNA, L-NAME and D-NAME on the perfusion of different tumours. The drugs were injected intravenously (20 mg $kg^{-1}$) into tumour-bearing mice and blood flow measurements from superficial tumour regions were made by laser Doppler flowmetry. Left graph shows individual blood flow traces from a single tumour recorded from L-NNA injected mice bearing either RIF (closed symbols) or FsaR tumours (open symbols). Right graph depicts average blood flow data from groups of L-NAME injected mice bearing either RIF (circles), SCCVII (diamonds) or EMT6 tumours (inverted triangles). Also shown are the results from RIF tumour-bearing mice injected D-NAME (squares). Bars, SD; *n* = 5.

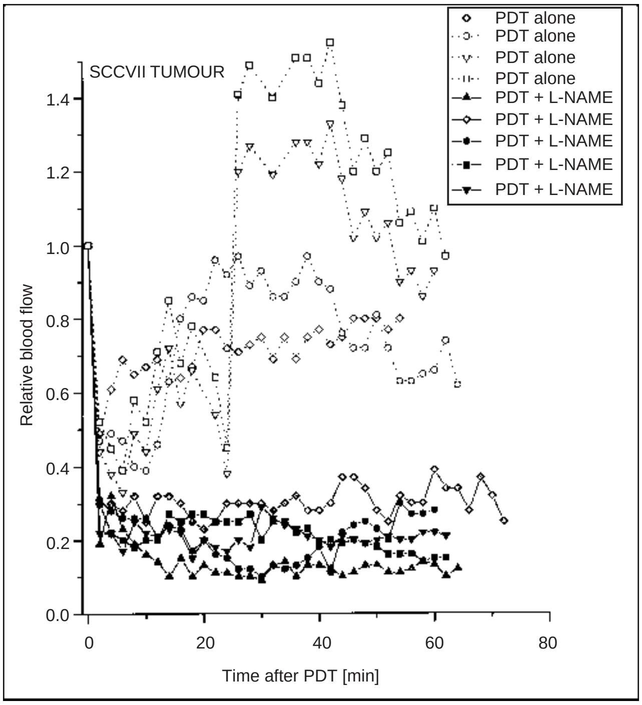

tumours, L-NAME exerted no apparent influence on the blood flow of the low NO producing EMT6 tumours. Also shown in the same graph (Figure 4, right) is the result obtained with D-NAME (RIF tumours), demonstrating that this metabolically inactive analogue of L-NAME is ineffective as a tumour blood flow inhibitor.

Since the effects of L-NAME on tumour blood flow correlate with its beneficial action on tumour response in combination with PDT, it was warranted to examine the effect of this drug on the blood flow of PDT-treated tumours. The PDT dose used in the experiments presented in Figure 3 promptly reduced the blood flow of treated SCCVII tumours to approximately 50% of normal levels, as shown with the examples of four individual tumours in Figure 5. However, this inhibitory effect generally lasted no longer than 20 minutes and then the blood flow was largely restored or

**Figure 5** The effect of L-NAME on the tumour blood flow changes induced by PDT. Mice bearing SCCVII tumours were given Photofrin (10 mg $kg^{-1}$ i.v.) followed 24 h later by 150 J $cm^{-2}$ of tumour-localized red light. Some of the mice were also injected L-NAME (20 mg $kg^{-1}$ i.v.) immediately after light treatment. Tumour blood flow was recorded before and after PDT as described in Figure 3. Individual traces are shown of four mice treated either by PDT alone (open symbols) or five mice treated by PDT plus L-NAME (closed symbols).

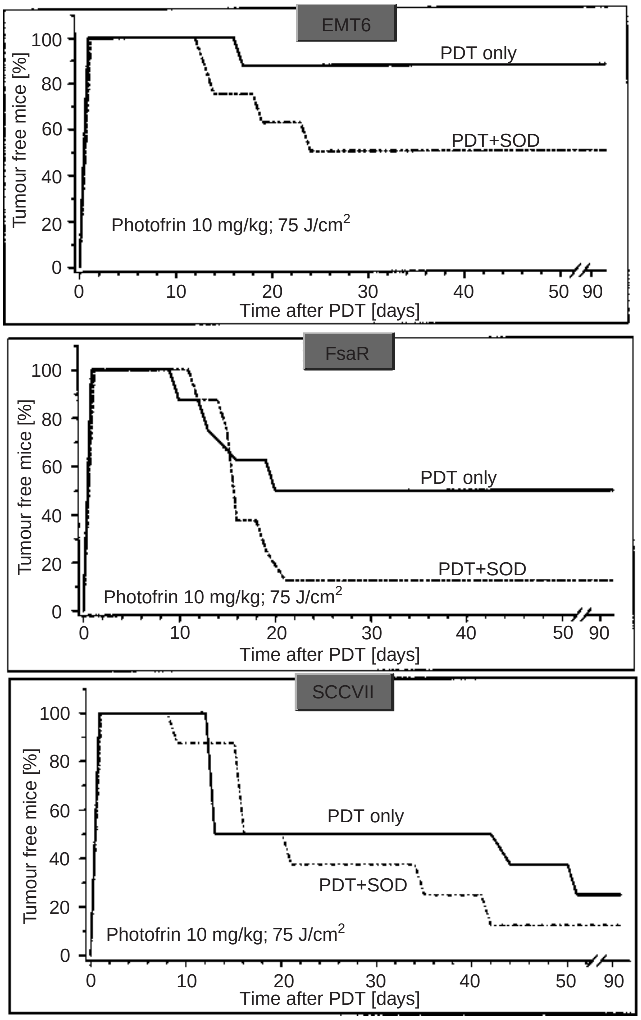

**Figure 6** The effect of superoxide dismutase (SOD) on the response of EMT6, FsaR and SCCVII tumours to PDT. Mice (eight per experimental group) bearing either EMT6 (top graph), FsaR (mid graph) or SCCVII tumour were treated by PDT as described in Figure 2, except that the light dose for SCCVII tumours was increased to 300 J $cm^{-2}$. SOD (450 U per mouse) was administered intravenously immediately after light treatment. Other details were as in Figure 2. The difference between the two treatment groups is statistically significant ($P < 0.05$) in the top two graphs.

even temporarily increased above normal levels in some tumours. In contrast, the blood flow in tumours of mice injected with L-NAME immediately after PDT was more profoundly reduced (to 10–30% of normal levels) and showed no signs of recovery up to the end of measurement (60–70 min post PDT); examples of five individual tumours are shown in Figure 5.

The observed pattern of changes in SCCVII tumour blood flow following PDT alone suggests that this treatment may induce ischaemia (as a consequence of the pronounced decrease in blood circulation) followed by reperfusion due to the restored normal blood flow. This can be associated with classical ischaemia-reperfusion injury, characterized by the production of superoxide in the treated tumour vasculature, which can contribute to the anti-tumour effect of PDT. To test this possibility, we have examined the effect of superoxide dismutase (SOD) on the tumour PDT response. The enzyme was injected intravenously immediately after the termination of PDT light treatment. The results with EMT6 and FsaR tumours show that the treatment with SOD markedly decreases the curative effect of PDT, while with SCCVII tumours this impact is much less pronounced and statistically insignificant (Figure 6). This finding corroborates the assumption that superoxide is formed after PDT and that the inhibition of its formation can be detrimental to the curative outcome of PDT, particularly with low NO-producing tumours. The inter-experimental variations in the PDT only response of EMT6 and FsaR tumours that can be noted in Figures 3 and 6 most likely originate from differences between cohort of tumours implanted at different times.

### DISCUSSION

In this study, we have used four mouse tumour models, two of which (RIF and SCCVII) are characterized by at least several-fold higher endogenous production of NO than the other two (EMT6 and FsaR). This qualification is based on the measurement of NO production using an in vitro assay in which tumour explants are fully oxygenated. This may not represent the actual in vivo tumour microenvironment characterized by the presence of variable sizes of hypoxic fraction in which NOS activity is arrested. Although showing marked heterogeneity, the hypoxic fraction is generally dependent of tumour size (Moulder and Rockwell, 1984), which in our experiments ranged 5–7 mm (largest diameter). At these sizes, hypoxic fractions may be large in EMT6 (> 30%), but not in SCCVII and FsaR (< 10%), and even less so in RIF tumours (< 2%) (Moulder and Rockwell, 1984; 1987). Thus, the size of the hypoxic fraction does not appear to determine our classification of tumours as high and low NO producers.

Alternate methods of NO measurement have other limitations. Probes inserted for in situ measurement damage blood vessels and may perturb tumour oxygenation, while immunohistochemistry assays do not quantify NOS activity in vivo. The advantage of the assay chosen in this study is that total NO production is tested under controlled conditions, containing fixed oxygenation and supply of co-factors, thereby allowing direct comparisons between tumour types. Our results show that the above classified two groups of tumours respond differently to the NO modulation treatments performed either alone or in conjunction with PDT.

In tumour models characterized by relatively high production of NO, lowering the NO levels immediately after PDT (by intravenously administered NOS inhibitors) appears to enhance the degree of destruction of treated tumours, as suggested by the improved tumour cure rates. Regular blood flow in these tumours appears to be maintained by a constant vasodilatation exerted through the presence of NO, as indicated by a strong impact of NOS inhibitors L-NNA and L-NAME (Figure 4). Moreover, NO has an important role in the restitution of PDT-inhibited tumour blood flow in those vessels that are not permanently damaged, since a more profound and longer lasting inhibition of tumour blood flow was observed with the L-NAME injection combined with PDT (Figure 5). On the other hand, tumours producing relatively low levels of NO (EMT6 and FsaR) are more sensitive to PDT, their blood perfusion is much less dependent on NO (showing very little, if any, sensitivity to the treatment with NOS inhibitors), and their PDT-mediated cure rate cannot be improved by NO level modulation. These results demonstrate that NO has an important role in events critical for the therapeutic outcome of PDT.

 *© 2000 Cancer Research Campaign*

Depending on tumour genotype and/or the level of its production, NO may play a facilitory or inhibitory role in tumour progression. The latter relates to the fact that high (micromolar) NO concentrations are cytotoxic (Schulz and Wambolt, 1995; Lala and Orucevic, 1998). The facilitory role NO exerts through the promotion of tumour blood flow (highly relevant for the effects investigated in this study), induction of angiogenesis in tumours (Fukumura and Jain, 1998; Lala and Orucevic, 1998) and promotion of tumour-cell invasiveness (Orucevic et al, 1999). In the present study, the modulation of NO levels achieved by a single treatment with NOS inhibitors was sufficient for a short-term modulation of tumour blood flow, but had no obvious impact on these other roles of NO in tumour progression, as evidenced by the absence of significant retardation of tumour growth (Figure 2).

Other authors (van Geel et al, 1994, 1996) already documented the induction of blood flow decrease by PDT in SCCVII and RIF tumour models. Results in our laboratory suggest that the intensity of this response is dependent on the PDT dose. The dose used in the experiments presented in Figure 5 is just below the curative threshold, and higher (curative) doses produce more pronounced or even permanent inhibition of blood flow in SCCVII and other tumour models (data not shown). However, even at relatively high PDT doses, vessel reperfusion may occur in tumour regions most distant to the source of illumination. The existence of episodes of partial or complete obstruction and subsequent restoration of tumour blood flow is of particular interest, because they may be associated with the induction of ischaemia-reperfusion injury (Kimura et al, 1996). During ischaemia, metabolic breakdown of ATP leads to the accumulation of xanthine/hypoxanthine and increased levels of xanthine oxidase (Grisham et al, 1986; Parkins et al, 1997). Consequently, a sudden re-introduction of oxygen at the time of reperfusion will result in a massive generation of superoxide in the affected tumour vasculature (Parkins et al, 1998). The presence of this oxygen radical in tumours following PDT was indirectly confirmed by the effect of SOD treatment on tumour cures (Figure 6). Since intravenously injected SOD is not likely to reach perivascular regions (or even endothelium), it can be concluded that a significant generation of oxygen radicals occurs in the vessel lumen of PDT-treated tumours. Earlier reports have also demonstrated the generation of superoxide in the skin of mice following PDT using ESR spectroscopy (Athar et al, 1988), and shown that the PDT effect can be inhibited by a SOD mimic compound or augmented by a SOD inhibitor (Athar et al, 1989).

The results of the experiments with SOD (Fig. 6) suggest that superoxide generated following tumour PDT treatment can contribute to the curative outcome both directly through inducing oxidative stress at the endothelium and indirectly through the interaction with NO. Because NO contains an unpaired electron and is paramagnetic, it rapidly reacts with superoxide forming peroxynitrite anion ($ONOO^-$) (Blough and Zalfiriou, 1985; McCall et al, 1989). Since very modest, if any, effect on PDT response is observed with high NO-producing SCCVII tumours, it may be speculated that under elevated NO concentrations superoxide is detoxified without reducing NO levels below the critical threshold. In contrast, under reduced NO concentrations present in EMT6 and FsaR tumours, NO levels may be further depleted through the interaction with PDT-generated superoxide, whereby superoxide may not be completely detoxified. Consequently, the administration of SOD following PDT treatment of these tumours results in scavenging of superoxide, which diminishes superoxide-mediated injury. Although it was suggested that SOD may act as a vascular modulator (Beckman et al, 1990), it is more likely that this effect is mediated through the interaction of superoxide with NO (Nakazono et al, 1991; Wolin, 1996).

Ischaemia-reperfusion insult is primarily inflicted by activated neutrophils massively invading the affected site (mediated through superoxide-induced upregulation of P-selectin) that induce considerable damage to the vasculature and surrounding tissue (Gaboury et al, 1994). The existence of a pronounced influx of neutrophils into PDT-treated tumours has been well-documented (Krosl et al, 1995; Gollnick et al, 1997; Korbelik and Cecic, 1998). Studies based on an experimental model of ischaemia-reperfusion injury induced by transiently clamping the feeding blood vessels to subcutaneous mouse tumours have demonstrated that this type of insult can produce significant generation of oxygen radicals leading to tumour cytotoxicity (Parkins et al, 1995; 1998). In these reports, it was also shown that NO exerts a strong protective role in this type of injury, since it affects more profoundly low NO-producing tumours and is potentiated by the L-NNA treatment. Endogenous NO production may also be one of the determinants of tumour sensitivity to PDT. This is indicated by the fact that EMT6 and FsaR tumours, ‘low’ NO producers, exhibit markedly greater sensitivity to PDT than ‘high’ NO producing RIF and SCCVII tumours. This property, if verified by more detailed experimental evidence, will deserve to be evaluated as a potential prognostic indicator for the outcome of PDT.

Further investigation needs to clarify which NOS isoforms (constitutional or inducible) are responsible for the endogenous NO production in chosen tumour models, and which cells are its main source. The PDT-induced changes in NO production in treated tumours have to be better characterized, particularly the NO-generating activity of neutrophils accumulating in PDT-treated lesions. As emphasized above, there are a number of events associated with PDT tumour responses that are influenced by the presence of NO. It can be assumed that, in addition to influencing blood flow and oxygen availability, NO controls the activity of platelets, neutrophils and other leukocytes, as well as the release of inflammatory mediators in the vasculature of PDT-treated tumours (Korbelik et al, 1998), and this merits further investigation.

### *Note added in proof*

After this paper was submitted for publication, a report was published by Henderson et al (1999). In this work, the authors show that the NOS inhibitor L-NNA, under conditions similar as in this study, affects the cure rate and blood flow in PDT-treated RIF tumours in a similar way as described in this report.

### ACKNOWLEDGEMENTS

Expert technical assistance was provided by Sandy Lynde. Photofrin (porfimer sodium) was provided by QLT Photo Therapeutics (Vancouver, Canada). This research is supported by the National Cancer Institute of Canada with funds from the Canadian Cancer Society (grant #008386).

### REFERENCES

Andrade SP, Hart IR and Piper PJ (1992) Inhibitors of nitric oxide synthase selectively reduce flow in tumour-associated neovasculature. *Br J Pharmacol* **107**: 1092–1095

© 2000 Cancer Research Campaign 

Athar M, Mukhtar H, Elmets CA, Zaim MT, Lloyd JR and Bickers DR (1988) *In situ* evidence for the involvement of superoxide anions in cutaneous porphyrin photosensitization. *Biochem Biophys Res Commun* **151**: 1054–1059

Athar M, Elmets CA, Bickers DR and Mukhtar H (1989) A novel mechanism for the generation of superoxide anions in hematoporphyrin derivative-mediated cutaneous photosensitization. Activation of xanthine oxidase pathway. *J Clin Invest* **83**: 1137–1143

Beckman JS, Beckman TW, Chen J, Marshall PA and Freeman BA (1990) Apparent hydroxyl radical production by peroxynitrite: implications for endothelial injury from nitric oxide and superoxide. *Proc Natl Acad SCI USA* **87**: 1620–1624

Blough NV and Zafiriou OC (1985) Reaction of superoxide with nitric oxide to form peroxynitrate in alkaline aqueous solution. *Inorganic Chemistry* **24**: 3504–3505

Bredt DS and Synder SH (1994) Nitric oxide: a physiologic messenger molecule. *Annu Rev Biochem* **63**: 175–195

Cecic I and Korbelik M (1999) Neutrophil-associated events in the inflammation of cancerous tissue following treatment with photodynamic therapy. *Trends in Photochemistry and Photobiology* (in press)

Dougherty TJ, Gomer CJ, Henderson BW, Jori G, Kessel D, Korbelik M, Moan J and Peng Q (1998) Photodynamic therapy. *J Natl Cancer Inst* **90**: 889–905

Durand RE and LePard NE (1994) Modulation of tumour hypoxia by conventional chemotherapeutic agents. *Int J Radiat Oncol Biol Phys* **29**: 481–486

Evans TJ, Buttery LDK, Carpenter A, Springall DR, Polak JM and Cohen J (1996) Cytokine-treated human neutrophils contain inducible nitric oxide synthase that produces nitration of ingested bacteria. *Proc Natl Acad Sci USA* **93**: 9553–9558

Fukumura D and Jain RK (1998) Role of nitric oxide in angiogenesis and microcirculation in tumors. *Cancer Metastasis Rev* **17**: 77–89

Gaboury JP, Anderson DC and Kubes P (1994) Molecular mechanisms involved in superoxide-induced leukocyte–endothelial cell interactions *in vivo*. *Am J Physiol* **266**: H637–H642

Gilissen MJ, van de Merbel-de Wit LE, Star WM, Koster JF and Sluiter W (1993) Effect of photodynamic therapy on the endothelium-dependent relaxation of isolated rat aortas. *Cancer Res* **53**: 2548–2552

Gollnick SO, Liu X, Owczarczak B, Musser DA and Henderson BW (1997) Altered expression of interleukin 6 and interleukin 10 as a result of photodynamic therapy *in vivo*. *Cancer Res* **57**: 3904–3909

Grisham MB, Hernandez LA and Granger DN (1986) Xanthine oxidase and neutrophil infiltration in intestinal ischaemia. *Am J Physiol* **251**: G567–G574

Gupta S, Ahmad N and Mukhtar H (1998) Involvement of nitric oxide during phthalocyanine (Pc4) photodynamic therapy-mediated apoptosis. *Cancer Res* **58**: 1785–1788

Hirst DG and Flitney FW (1997) The physiological importance and therapeutic potential of nitric oxide in tumour-associated vasculature. In *Tumour Angiogenesis*, Bicknell R, Lewis CE, Ferrara N (eds), pp. 153–167. Oxford University Press: Oxford

Henderson et al (1999) *Photochem Photobiol* **70**: 64–71

Horsman MR, Chaplin DJ, Hill SA, Arnold S, Collingridge D, Radacic M, Wood PJ, and Overgaard J (1996) Effect of nitro-L-arginine on blood flow, oxygenation and the activity of hypoxic cell cytotoxins in murine tumours. *Br J Cancer* **74**: S168–S171.

Jenkins DC, Charles IG, Thomsen LL, Moss DW, Holmes LS, Baylis SA, Rhodes P, Westmore K, Emson PC and Moncada S (1995) Roles of nitric oxide in tumour growth. *Proc Natl Acad Sci USA* **92**: 4392–4396

Kimura H, Braun RD, Ong ET, Hsu R, Secomb TW, Papahadjopoulos D, Hong K and Dewhirst MW (1996) Fluctuations in red cell flux in tumor microvessels can lead to transient hypoxia and reoxygenation in tumor parenchyma. *Cancer Res* **56**: 5522–5528

Korbelik M, Cecic I and Shibuya H (1997) The role of nitric oxide in the response of cancerous lesions to photodynamic therapy. *Recent Res Devel Photochem & Photobiol* **1**: 267–276

Korbelik M and Krosl G (1996a) Photofrin accumulation in malignant and host cell populations of various tumours. *Br J Cancer* **73**: 506–513

Korbelik M and Krosl G (1996b) Photosensitizer distribution and photosensitized damage of tumour tissues. In: *The Fundamental Bases of Phototherapy*, Hönigsman H, Jori G, Yang AR (eds), pp. 229–245. OEMF spa: Milan

Korbelik M and Cecic I (1998) Enhancement of tumour response to photodynamic therapy by adjuvant mycobacterium cell-wall treatment. *J Photochem Photobiol B: Biol* **44**: 151–158

Korbelik M, Shibuya H and Cecic I (1998) Relevance of nitric oxide to the response of tumors to photodynamic therapy. *Proc SPIE* **3247**: 98–105

Krosl G, Korbelik M and Dougherty GJ (1995) Induction of immune cell infiltration into murine SCCVII tumour by Photofrin-based photodynamic therapy. *Br J Cancer* **71**: 549–555

Kubes P, Suzuki M and Granger DN (1991) Nitric oxide: an endogenous modulator of leukocyte adhesion. *Proc Natl Acad Sci USA* **88**: 4651–4655

Lala PK and Orucevic A (1998) Role of nitric oxide in tumor progression: lessons from experimental tumors. *Cancer Metastasis Rev* **17**: 91–106

McCall TB, Boughton-Smith NK, Palmer RM, Whittle BJ and Moncada S (1989) Synthesis of nitric oxide from L-arginine by neutrophils. Release and interaction with superoxide anion. *Biochem J* **261**: 293–296

Mehta JL, Lawson DL, Nicolini FA, Ross MH and Player DW (1991) Effects of activated polymorphonuclear leukocytes on vascular smooth muscle tone. *Am J Physiol* **261**: H327–H334

Moilanen E, Vuorinen P, Kankaanranta H, Metsä-Ketelä T and Vapaatalo H (1993) Inhibition by nitric oxide-donors of human polymorphonuclear leucocyte functions. *Br J Pharmacol* **109**: 852–858

Moulder JE and Rockwell S (1984) Hypoxic fractions of solid tumors: experimental techniques, methods of analysis, and a survey of existing data. *Int J Radiat Oncol Biol Phys* **10**: 695–712

Moulder JE and Rockwell S (1987) Tumor hypoxia: its impact on cancer therapy. *Cancer Metastasis Rev* **5**: 313–341

Nakazono K, Watanabe N, Matsuno K, Sasaki J, Sato T and Inoue M (1991) Does superoxide underlie the pathogenesis of hypertension? *Proc Natl Acad Sci USA* **88**: 10045–10048

Ochsner M (1997) Photophysical and photobiological processes in the photodynamic therapy of tumours. *J Photochem Photobiol B: Biol* **39**: 1–18

Orucevic A, Bechberger J, Green AM, Shapiro RA, Billiar TR and Lala PK (1999) Nitric-oxide production by murine mammary adenocarcinoma cells promotes tumor-cell invasiveness. *Int J Cancer* **81**: 889–896

Parkins CS, Dennis MF, Stratford MRL, Hill SA and Chaplin DJ (1995) Ischemia reperfusion injury in tumors: the role of oxygen radicals and nitric oxide. *Cancer Res* **55**: 6026–6029

Parkins CS, Hill SA, Stratford MR, Dennis MF and Chaplin DJ (1997) Metabolic and clonogenic consequences of ischaemia reperfusion insult in solid tumours. *Exp Physiol* **82**: 361–368

Parkins CS, Holder AL, Dennis MF, Stratford MR and Chaplin DJ (1998) Involvement of oxygen free radicals in ischaemia-reperfusion injury to murine tumours: role of nitric oxide. *Free Radic Res* **28**: 271–281

Rees DD, Cunha FQ, Assreuy J, Herman AG and Moncada S (1995) Sequential induction of nitric oxide synthase by *Corynebacterium parvum* in different organs of the mouse. *Br J Pharmacol* **114**: 689–693

Rockwell SC, Kallman RF and Fajardo LF (1972) Characteristics of a serially transplanted mouse mammary tumor and its tissue-culture-adapted derivative. *J Natl Cancer Inst* **49**: 735–749

Schmidt HHH and Walter U (1994) NO at work. *Cell* **78**: 919–925

Schulz R and Wambolt R (1995) Inhibition of nitric oxide synthesis protects the isolated working rabbit heart from ischaemia-reperfusion injury. *Cardiovasc Res* **30**: 432–439

Shepherd AP, Riedel GL, Kiel JW, Haumschild DJ and Maxwell LC (1987) Evaluation of an infrared laser Doppler blood flowmeter. *Am J Physiol* **252**: G832–G839

Stratford MRL, Dennis MF, Cochrane R, Parkins CS and Everett SA (1997) The role of nitric oxide in cancer: improved methods for measurement of nitrite and nitrate by high-performance ion chromatography. *J Chromatogr* **770**: 151–155

Suit HD, Sedlacek RS, Silver G and Dosoretz D (1985) Pentobarbital anesthesia and response of tumor and normal tissue in the C3Hf/Sed mouse to radiation. *Radiat Res* **104**: 47–65

Tozer GM and Everett SA (1997a) Nitric oxide in tumour biology and cancer therapy. Part 2: therapeutic implications. *Clin Oncol* **9**: 357–364

Tozer GM and Everett SA (1997b) Nitric oxide in tumour biology and cancer therapy. Part 1: physiological aspects. *Clin Oncol* **9**: 282–293

Twentyman PR, Brown JM, Gray JW, Franko AJ, Scoles MA and Kallman RF (1980) A new mouse tumor model system (RIF-1) for comparison of end-point studies. *J Natl Cancer Inst* **64**: 595–604

van Geel IPJ, Oppelaar H, Oussoren YG and Stewart FA (1994) Changes in perfusion of mouse tumours after photodynamic therapy. *Int J Cancer* **56**: 224–228

van Geel IPJ, Oppelaar H, Rijken PFJW, Bernsen HJJA, Hagemeier NEM, van der Kogel AJ, Hodgkiss RJ and Stewart FA (1996) Vascular perfusion and hypoxic areas in RIF-1 tumours after photodynamic therapy. *Br J Cancer* **73**: 288–293

Vanhoutte PM (1987) Endothelium and responsiveness of vascular smooth muscle. *J Hypertens Suppl* **5**: S115–S120

Volpe JP, Hunter N, Basic I and Milas L (1985) Metastatic properties of murine sarcomas and carcinomas. I. Positive correlation with lung colonization and lack of correlation with s.c. tumor take. *Clin Exp Metastasis* **3**: 281–294

Wolin MS (1996) Reactive oxygen species and vascular signal transduction mechanisms. *Microcirculation* **3**: 1–17

 *© 2000 Cancer Research Campaign*

Wood PJ, Sansom JM, Butler SA, Stratford IJ, Cole SM, Szabo C, Thiemerman C and Adams GE (1994) Induction of hypoxia in experimental murine tumors by the nitric oxide synthase inhibitor, $N^G$-nitro-L-arginine. *Cancer Res* **54**: 6458–6463

Xie K, Huang S, Dong Z, Gutman M and Fidler IJ (1995) Direct correlation between expression of endogenous inducible nitric oxide synthase and regression of M5076 reticulum cell sarcoma hepatic metastases in mice treated with liposomes containing lipopeptide CGP 31362. *Cancer Res* **55**: 3123–3131

© 2000 Cancer Research Campaign
